# Impact of meteorological variability on diurnal and seasonal net ecosystem productivity in a desert riparian forest ecosystem

**DOI:** 10.3389/fpls.2024.1332192

**Published:** 2024-04-18

**Authors:** Dexiong Teng, Xuewei Gong, Xuemin He, Jingzhe Wang, Guanghui Lv, Jinlong Wang, Xiaodong Yang

**Affiliations:** ^1^ CAS Key Laboratory of Forest Ecology and Management, Institute of Applied Ecology, Chinese Academy of Sciences, Shenyang, China; ^2^ College of Ecology and Environment, Xinjiang University, Urumqi, China; ^3^ Xinjiang Jinghe Observation and Research Station of Temperate Desert Ecosystem, Ministry of Education, Urumqi, China; ^4^ School of Artificial Intelligence, Shenzhen Polytechnic University, Shenzhen, China; ^5^ Department of Geography & Spatial Information Technology, Ningbo University, Ningbo, China

**Keywords:** arid ecosystem, eddy covariance, carbon flux, circadian regulation, structure equation model

## Abstract

The desert riparian forests are susceptible to meteorological changes and contribute significantly to the net ecosystem productivity (NEP) variations of arid ecosystems. However, the responsive patterns of their NEP variations to the meteorological variabilities remain inadequately comprehended. To address this gap, we utilized seven years of eddy covariance flux measurements in a representative desert riparian forest to investigate the NEP variations and its response to changing meteorological factors across diverse temporal scales. The results revealed significant periodic variations in half-hourly NEP, with dominant cycles spanning from five hours to one year, with a principal oscillation period of one day. Key meteorological factors including global solar radiation (Rg), relative humidity (RH), air temperature (Ta), soil temperature (Ts), and vapor pressure deficit (VPD) exhibited synchronization with NEP on daily scales. This synchronization, coupled with the observed one-day periodic NEP variations, provides robust evidence supporting the existence of a circadian rhythm in the ecosystem carbon exchange of desert riparian forest regulated by meteorological conditions. Seasonal patterns were significant in the impact of Rg phase, Ta diurnal amplitude, and VPD diurnal amplitude on NEP diurnal amplitude and phase. The NEP diurnal amplitude significantly, directly, and positively affected daily NEP in both the dormant and growing seasons, whereas its phase yielded significant negative effects (*P*< 0.05). The averages, amplitudes, and phases of diurnal meteorological conditions controlled the daily NEP by regulating NEP diurnal amplitude and phase. These findings provide evidence that the variability in circadian rhythms, caused by the increase in diurnal Ta and VPD, significantly impact the daily NEP at an ecosystem scale. This study enriches our comprehension of the meteorological mechanisms governing diurnal and seasonal carbon uptake dynamics within desert riparian forests, providing fresh insights into the direct and indirect roles of climate change in shaping patterns of ecosystem carbon exchange.

## 1 Introduction

Net ecosystem productivity (NEP) measures the equilibrium between carbon uptake through photosynthesis and carbon loss via respiration, serving as a critical indicator of ecosystem carbon sequestration and stability. Variations in NEP can reflect ecosystem responses and adaptability to ongoing climate change. Arid and semi-arid ecosystems constitute about 30% of the Earth’s land area and play a substantial role in the interannual variability of the global terrestrial NEP, accounting for 39% of this process (Ahlstrom et al., 2015). The Earth’s land surface has experienced significant warming and an increase in atmospheric vapor pressure deficit (VPD) over the past century, particularly noting a greater rise in daily minimum than maximum temperature in many regions (Thorne et al., 2016; Yuan et al., 2019), and including the most of arid and semi-arid lands. The pronounced trend underscores the urgency for understanding the impact of climate change on NEP in these vulnerable ecosystems. However, the mechanisms whereby climate change impacts NEP in these ecosystems have not been completely comprehended. Climate change alters meteorological patterns at regional and global scales, which may significantly impact the NEP (Reichstein et al., 2013; Franklin et al., 2016; Tang et al., 2022). To comprehensively understand and accurately predict the NEP’s response to climate change, an exigency exists to investigate the impact of meteorological changes at varying time scales on the NEP.

The elucidation of NEP from a meteorological perspective begins with transformations in key driving factors governing the carbon uptake process, including solar radiation, air temperature (Ta), atmospheric humidity, VPD, and soil moisture (Law et al., 2002; van Dijk et al., 2005; Yi et al., 2010). Previous investigations have mainly focused on the direct impacts of meteorological factors on NEP. For instance, excessive light during photosynthesis can induce photoinhibition in plants (Chaves, 1991), and solar radiation determined the NEP in deserts (Yu et al., 2023). Significant depletion of humidity and soil moisture led to stomatal closure and diminished photosynthetic activity (Reichstein et al., 2007; Wolf et al., 2016). Half-hour photosynthesis and respiration exhibited a nonlinear response to temperature changes (Baldocchi et al., 2001; Way and Yamori, 2013). A synchronization mechanism governed by light and temperature regulated the timing of photosynthesis and stomatal aperture in plants (Masuda et al., 2021). Although these outstanding scientific works support the direct impact of meteorological factors, only a few studies have explored their indirect impact on NEP changes, most of which disregarded the influences of amplitude and phase of the meteorology fluctuations. In contrast, these variations in meteorology (such as light, temperature, and humidity) have been suggested to affect the circadian rhythms of tree physiology (Way and Montgomery, 2015; Singh et al., 2021). The circadian rhythms of tree physiological processes, such as leaf stomatal conductance and photoperiodic responses with a period of ∼24 h, were important drivers of the photosynthesis and respiration in ecosystems, although they required the passage of a few hours (Resco de Dios and Gessler, 2018). Additionally, the amplitude of diurnal NEP changes under constant environmental conditions was 20% to 90% of that under variable environmental conditions (Dios et al., 2012). The diel and seasonal dynamics of stem growth of trees at the community level were significantly influenced by temperature and VPD (Zhou et al., 2023). The diel patterns in carbon flux were expected to be the key factors in understanding stem growth (Steppe et al., 2015). On one hand, the control and manipulation of meteorological conditions at the ecosystem level for direct tests of circadian regulation response to short-term meteorological changes pose a significant challenge. On the other hand, the variation in NEP under variable conditions may be caused by several endogenous and exogenous mechanisms. Up until now, however, there has been a knowledge gap regarding how the amplitude and phase of diurnal variations in meteorology affect the NEP of arid and semi-arid ecosystems.

Desert riparian forests played a critical role in arid and semi-arid ecosystems due to their remarkable ecosystem biodiversity and carbon sequestration (DéCamps et al., 2004; Thevs et al., 2011). Tugai forests (*Populus euphratica* forests) were widely distributed in the natural oasis of the lower inland rivers and constitute the largest types of natural desert riparian forests in arid regions (Feng and Cheng, 1998). The area of these forests in China was approximately 6.49 × 10^5^ hm^2^, constituting 92.3% of the entire desert riparian forest area in the country (Ding et al., 2017; Wang et al., 2017; Fan et al., 2018). Due to being subjected to the severe environmental stress such as drought and salinity, Tugai forests were particularly vulnerable to variations in the meteorological environment (Jiang et al., 2022). However, their above-ground carbon density ranged from 2.24 t/hm^2^ to 30.42 t/hm^2^, giving them a greater carbon sink potential than other arid ecosystems such as grasslands and sparse shrublands (Liu et al., 2016). Hence, Tugai forests are ideal for studying the effects of changing meteorological conditions on the NEP of desert riparian forest ecosystems.

Utilizing approximately seven years of eddy covariance flux measurements in a representative Tugai forest, this study introduced the amplitudes and phase of daily variations to examine the direct and indirect response mechanisms of NEP to changing meteorological conditions in both the growing and dormant seasons. The objectives of this study are to explore the variation patterns of NEP and meteorological conditions in the desert riparian forests at different time scales, analyze the relationship between NEP and meteorological conditions at various time scales, and investigate the direct and indirect response mechanisms of NEP to the changes in meteorological conditions at the daily scale. This study is expected offers insights into understanding the processes of the forest ecosystems carbon cycle, thereby enhancing predictions of carbon exchange within these ecosystems under the ongoing context of climate change.

## 2 Materials and methods

### 2.1 Site description

The study was conducted at the Ebinur Lake Wetland National Nature Reserve in northwestern Xinjiang, China, from January 1, 2012 to April 20, 2019. The study area (44°37′05″−45°10′35″N, 82°30′47″−83°50′21″E) experiences a north temperate continental arid climate, characterized by hot summers, ample sunlight, low precipitation, and cold winters. The distribution of annual precipitation is uneven, with more in summer (~50 mm) and less in winter (<10 mm). The long-term average precipitation is 105.17 mm, and the evaporation is 1315 mm (Li et al., 2022). The flux tower was positioned approximately 100 m from the north bank of the Aqikesu River (44°37′4.8″N, 83°33′59.4″E) within the nature reserve, with a construction height of 33 m (Teng et al., 2021). There are over 30 species of plants in the study area (Zhang et al., 2015). Species such as *Populus euphratica*, *Haloxylon ammodendron* and *Phragmites australis* are dominant within investigated riparian vegetation and represent over then 60% of total vegetation coverage in the studied area. Additionally, there are a variety of halophytic shrubs, herbs, and desert-specific short-growing plants, such as *Halimodendron halodendron*, *Halocnemum strobilaceum* and *Suaeda glauca*, with an average community canopy height of approximately 8.5 m (He et al., 2014). The soil has high salinity and alkalinity, with an average electrical conductivity of 5.41 mS/cm in shallow soil layer (0–10 cm) and a pH value of 8.77. The average soil density is about 1.38 g/cm^3^ (Li et al., 2022). Throughout the observation period, the flux tower was located in an area with an average annual temperature of 9°C, with maximum temperatures reaching 43°C and minimum temperatures dropping to −26°C. Groundwater served as the primary water source for the plants in the Tugai forest ecosystem, with a groundwater depth of 1.50-2.30 m (Yang et al., 2014).

### 2.2 Eddy covariance and meteorological measurements

The eddy covariance (EC) observation system was positioned 15 m above the ground. It comprises a three-dimensional ultrasonic anemometer (CSAT3, Campbell Scientific Ltd., Logan, UT, USA) and an infrared CO_2_/H_2_O analyzer (EC150, Campbell Scientific Ltd., Logan, UT, USA). Global radiation was monitored using a 4-component net radiometer (NR01, Campbell Scientific Ltd., Logan, UT, USA) installed at a height of 9 m. Additionally, the meteorological observation system includes air temperature and humidity sensors (HMP155A-L, Campbell Scientific Ltd., Logan, UT, USA), wind speed sensors (010C-1, Met One Instruments Inc., Grants Pass, OR, USA), wind direction sensors (020C-1, Met One Instruments Inc., Grants Pass, OR, USA), and an atmospheric pressure meter (CS100, Campbell Scientific Ltd., Logan, UT, USA). These instruments are programmed to automatically record routine meteorological data, such as average wind speed, temperature, and air pressure every 30 minutes.

### 2.3 Flux calculations and data processing

#### 2.3.1 Flux calculation

Assuming horizontal homogeneity, the net ecosystem CO_2_ exchange (NEE) in μmol m^−2^ s^−1^ can be estimated as follows (Baldocchi, 2003; Wang et al., 2016):


(1) NEE=Fc+Fs=ρd¯·w′χc'¯│h+Δχc·ρd¯·hΔt.

In Equation 1, $F_{c}$ represents the turbulent flux at the interface between the atmosphere and the ecosystem. $F_{s}$ corresponds to the CO_2_ storage flux within the control volume. w stand for the vertical wind speed. $\chi_{c}$ denotes the CO_2_ molar mixing ratio (μmol mol^−1^). $\rho_{d}$ represents the dry air concentration (mol m^−3^). *h* is the flux observation height (m); $\text{Δ}\chi_{c}$ indicates the difference between the pre- and post- CO_2_ molar mixing ratio measured at two adjacent moments. Δt is the time interval between the first and second measurements (30 min).

The turbulent fluxes (F_c_) were calculated from high-frequency measurements. Raw data files were processed using EddyPro software (Version 6, LI-COR Inc., Lincoln, NE, USA) to calculate the F_c_ on a half-hourly basis. The flux processing included two-dimensional coordinate rotation, spectral corrections, frequency response correction, and Webb-Pearman-Leuning correction for the effect of air density fluctuations (Webb et al., 1980). The quality assessment for F_c_ data were conducted a steady-state/developed turbulence conditions test in 0-2 system (Mauder et al., 2006). The CO_2_ storage (F_s_) was estimated using the single-point method (Hollinger et al., 1994).

#### 2.3.2 Data processing

The NEP is analogous to net photosynthesis in leaves, and is considered positive when the ecosystem exhibits net carbon uptake, indicating a carbon sink. It is the opposite of NEE (Lovett et al., 2006), that is NEP=−NEE . As ecosystem carbon exchange processes are highly dependent on diurnal rhythms and phenological laws, we identified the beginning and end of the day and the beginning and end of the growing season. The solar elevation angle was used to define the diurnal period as from sunrise to sunset. As presented in **Table 1**, the start and end of the growing season were determined based on the growing degree days (GDDs), and the formula was as follows (McMaster and Wilhelm, 1997):

**Table 1:** The starting and ending date of growing season at observation stations.

Year	Start (DOY)	End (DOY)	Duration (Day)
2012	113	283	171
2013	99	290	192
2014	120	279	160
2015	110	286	177
2016	119	283	165
2017	120	275	156
2018	99	284	186
2019	104		


(2) $$GDD=\frac{1}{2}\left(T_{max}+T_{min}\right)-T_{base},$$

In Equation 2, T_base_ was 6°C in this study.

The gaps in carbon flux measurements amounted to 59,906 half-hour data (approximately 46.52% of the total), and these gaps were primarily concentrated during the dormant season, especially during nighttime hours. Surprisingly, the number of data gaps during nighttime in the dormant season was more than twice as much as during nighttime in the growing season. Conversely, the high-quality data (Qc=0) accounted for 35,852 half-hour data (approximately 27.84% of the total), and were predominantly record during the daytime in the growing season. Remarkably, the amount of high-quality flux data during nighttime in the growing season was comparable to that during nighttime in the dormant season (**Table 2**).

**Table 2:** Statistics on half-hour carbon flux data.

	Growing season daytime	Growing season nighttime	Dormant season daytime	Dormant season nighttime	Total
NA	13750	8817	15449	21890	59906
Qc=0	14765	6833	8221	6033	35852
Qc=1	4286	5416	4822	7807	22331
Qc=2	1728	2554	2225	4077	10584
Total	34529	23620	30817	39807	128773

In this study, distinct frictional velocity (u_*_) thresholds and their corresponding confidence intervals were estimated separately for the growing and dormant seasons, considering various wind direction intervals (30°). The carbon flux data underwent rigorous screening based on the criteria outlined in **Table 3**, leading to the exclusion of any data that failed to meet these conditions. Finally, the gap-filling procedure utilized the random forest model, achieving an impressive R^2^ value of 0.81.

**Table 3:** Quality control process of carbon flux data.

Procedure	Volume of data		Detailed description
	Growing season	Dormant season	
Quality assessment	22567	37339	Based on the quality assessment of flux data, carbon flux data with null values and Qc of 2 were rejected.
Weather anomalies	36	152	Carbon flux data from precipitation over the observation period were excluded.
Mutation filtering	121	157	The mutation points in continuous flux data were detected and rejected.
Statistical distribution cleaning	360	468	Outliers in the flux data, counted at different times of the day, were eliminated.
u_*_ threshold filtering	3349	3575	Nighttime observations below the u_*_ thresholds were excluded.

#### 2.3.3 Amplitude and phase of the variations in NEP and meteorological conditions

**Figure 1:**
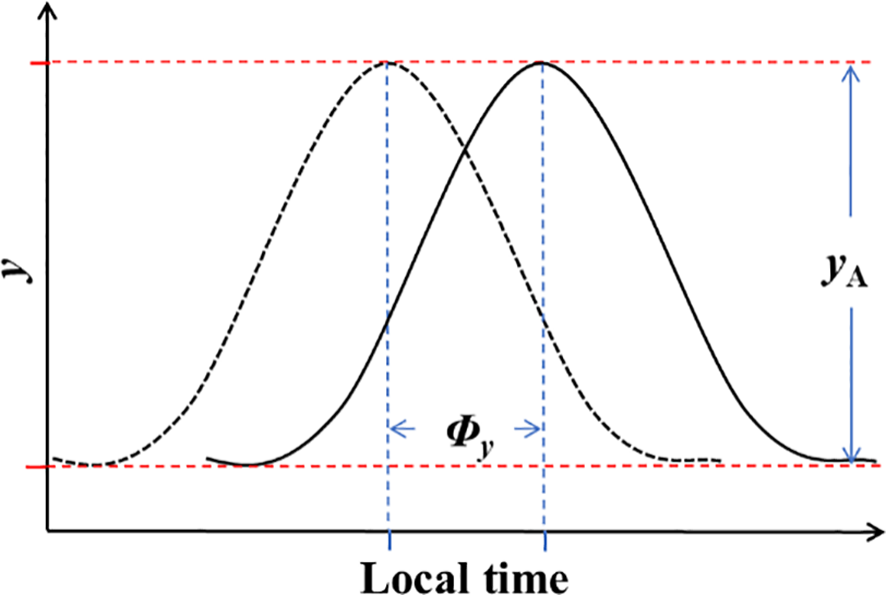
The regulation of NEP and meteorological conditions by amplitude (*y*_A_) and phase (*Φ_y_*) on a daily scale.


To comprehensively describe the diurnal variations in NEP and meteorological factors, two key parameters were computed: amplitude and phase. Circadian regulation is known to result in time changing maximal and minimal potential values, as depicted in **Figure 1**. Assuming that circadian effects have an additive interaction with the mean value of the parameter of interest (Resco de Dios and Gessler, 2018), the models for circadian variations can be expressed as follows:

(3) $$y=y_{m}+y_{A}\sin\left(\frac{2\pi t}{24}+\Phi_{y}\right).$$

In Equation 3: *y* represents NEP or meteorological factors; $y_{m}$ denotes the mean value of *y*; *t* stands for time; and $y_{A}$ and $\Phi_{y}$ represent the amplitude and phase of half-hourly NEP or meteorological factors variations in a daily scale, respectively. The variation in amplitude signifies the asymmetric changes in meteorological factors and the differences in diurnal and nocturnal variations in NEP (where daytime increases or decreases differ in magnitude from nighttime). On the other hand, the variation in phase denotes the changes in the timing of daily maximum or minimum values. These two parameters offered a clearer understanding of the daily patterns of carbon uptake and meteorological changes in the ecosystem.

### 2.4 Statistical analysis

#### 2.4.1 Magnitude squared coherence and transfer function

The Fourier transform was applied to the time series of NEP and meteorological factors, and subsequently, the power spectrum of the time series is calculated and analyzed in terms of magnitude-squared coherence (MSC) and transfer function variation periods. MSC, a signal processing tool that yields a real value ranging from zero to one, is employed to identify significant frequency-domain correlations between two time series (Dobie and Wilson, 1989). It quantifies the degree to which two time-domain signals, *x*(*t*) and *y*(*t*), exhibit similarity or match each other.

To estimate the synchronization of two time series, the linear correlation in the spectral decomposition of *x*(*t*) and *y*(*t*) is followed by calculating the *MSC_xy_* (*f*) values at different frequencies using the equation:


(4) $$MSC_{xy}\left(f\right)=\frac{{\left|P_{xy}\left(f\right)\right|}^{2}}{P_{xx}\left(f\right)P_{yy}\left(f\right)},\;$$

In Equation 4, $P_{xy}$ is the cross power spectral density of *x*(*t*) and *y*(*t*), and $P_{xx}$ and $P_{yy}$ are the associated power spectral densities.

In this study, Welch’s mean-corrected periodogram method is utilized to calculate the power and mutual power spectral densities between *x*(*t*) and *y*(*t*). This method involves dividing the two signals into time windows with the same number of samples, calculating the power spectral density for each window, and then averaging them to obtain the final *MSC_xy_* (*f*) values at different frequencies.

A transfer function is a complex quantity whose magnitude and phase are a function of frequency. Referred to as the system function, it describes the transfer behavior of a linear system in the frequency domain, with the output being represented by *H*(*t*) and the input represented by the z-transform of the impulse response *H*(z).

The transfer function is given by the equation:


(5) $$H\left(z\right)=\frac{Y\left(z\right)}{X\left(z\right)}\;,$$

In Equation 5, *Y*(*z*) represents the output transformation and *X*(*z*) represents the input transformation. The transfer function *H*(*z*) captures the transfer characteristics of the system, as it multiplies the input transformation *X*(*z*) to obtain the output transformation *Y*(*z*). The transfer function can be derived from simple algebraic operations that describe the differential equations of the system, or it can be determined experimentally to understand the transfer behavior of the system.

#### 2.4.2 Structural equation model

Structural equation model (SEM), including path analysis, confirmatory factor analysis, and latent growth curve models, is primarily employed to examine multivariate interactions (McIntosh et al., 1996). As a relatively complex statistical model, SEM requires a sample size of at least 200 to yield reliable results (Fan et al., 1999). This is due to the fact that more intricate models necessitate larger samples to achieve statistical power. Furthermore, due to certain mathematical restrictions that limit the complexity of the multivariate model, a balance must be struck between model complexity, precision, and interpretability when employing SEM. We use path analysis to analyze the covariance between NEP and observed variables, with the structure of the model being driven by assumptions about causal relationships between multiple variables. For all statistical analyses, we used R version 4.2.0 (R Core Team, 2022).

## 3 Results

### 3.1 Environmental conditions and net ecosystem production

**Figure 2:**
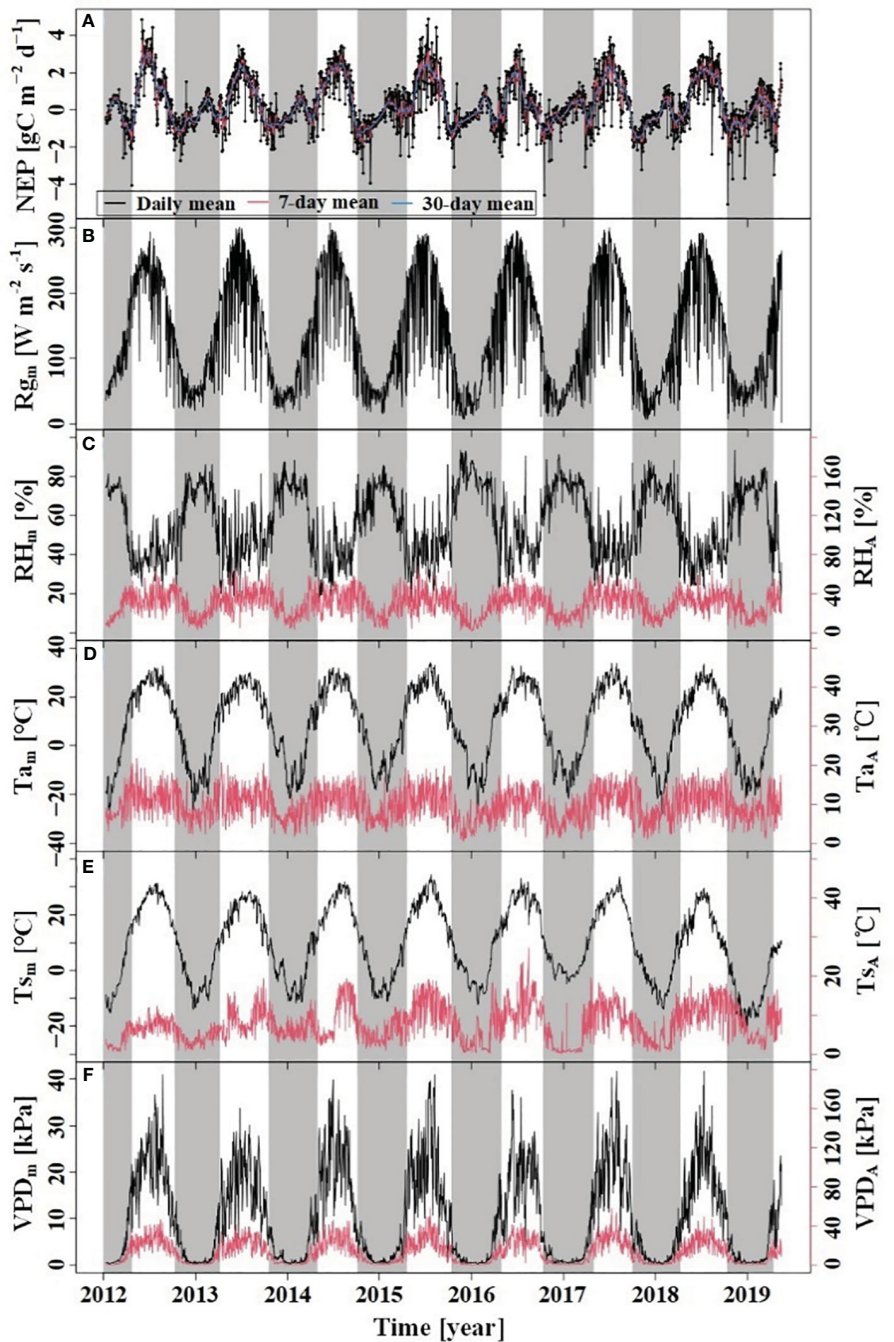
Variations of NEP and meteorological factors. **(A)** NEP: net ecosystem productivity; **(B) **Rg_m_: daily mean of Rg; **(C) **RH_m_: daily mean RH, RH_R_: amplitude of diurnal RH; **(D)** Ta_m_: daily mean Ta, TaR: amplitude of diurnal Ta; **(E)** Tsm: daily mean Ts, TsR: amplitude of diurnal Ts; **(F)** VPDm: daily mean VPD, VPD_R_: amplitude of diurnal VPD. The grey region indicates the dormant season and the white indicates the growing season.

During the study period, the dynamics of Ta_m_, Ts_m_, VPD_m_, and Rg_m_ above the forest canopy exhibited a symmetrical pattern between the growing and dormant seasons (**Figure 2**). Notably, Rg_m_, VPD_m_, Ta_m_, and Ts_m_ showed significant seasonal variations, with higher values recorded in the growing season and lower values in the dormant season. During the dormant season, the RH_m_ was higher than compared to the growing season. The Ta_m_ during the study period was 8.83°C, with a range of variation from −28.04°C to 34.13°C; VPD_m_ ranged from 0.02 kPa to 4.17 kPa, with the maximum and minimum values observed in July and January, respectively.

**Figure 3:**
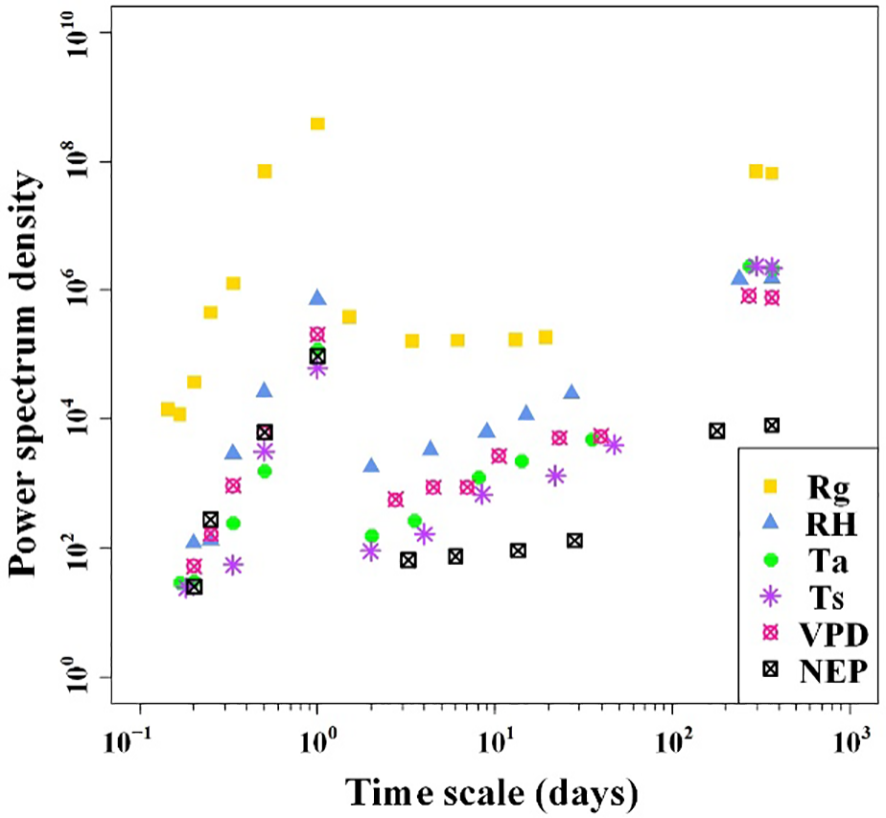
Power spectrum densities of half-hourly NEP and meteorological factors over the duration of monitoring.

The results of the spectral analysis performed on the NEP and meteorological factor time series of the Tugai forest were presented in **Figure 3**. It is evident that both NEP and meteorological factors exhibit multiple periodicities, ranging from five hours to one year. Except for Rg, the amplitudes of fluctuations in both NEP and meteorological factors also increased when the period length exceeded one day but remained less than 40 days. When the periods of variability in NEP and meteorological factors were equal to or less than one day, the amplitude of fluctuations tended to become more pronounced with increasing period length. The NEP variations exhibited the strongest correlation with meteorological conditions in the one-day cycle. This observation implied that NEP in the Tugai forest followed a circadian rhythm.

### 3.2 Interactions between net ecosystem production and environmental variation

**Figure 4:**
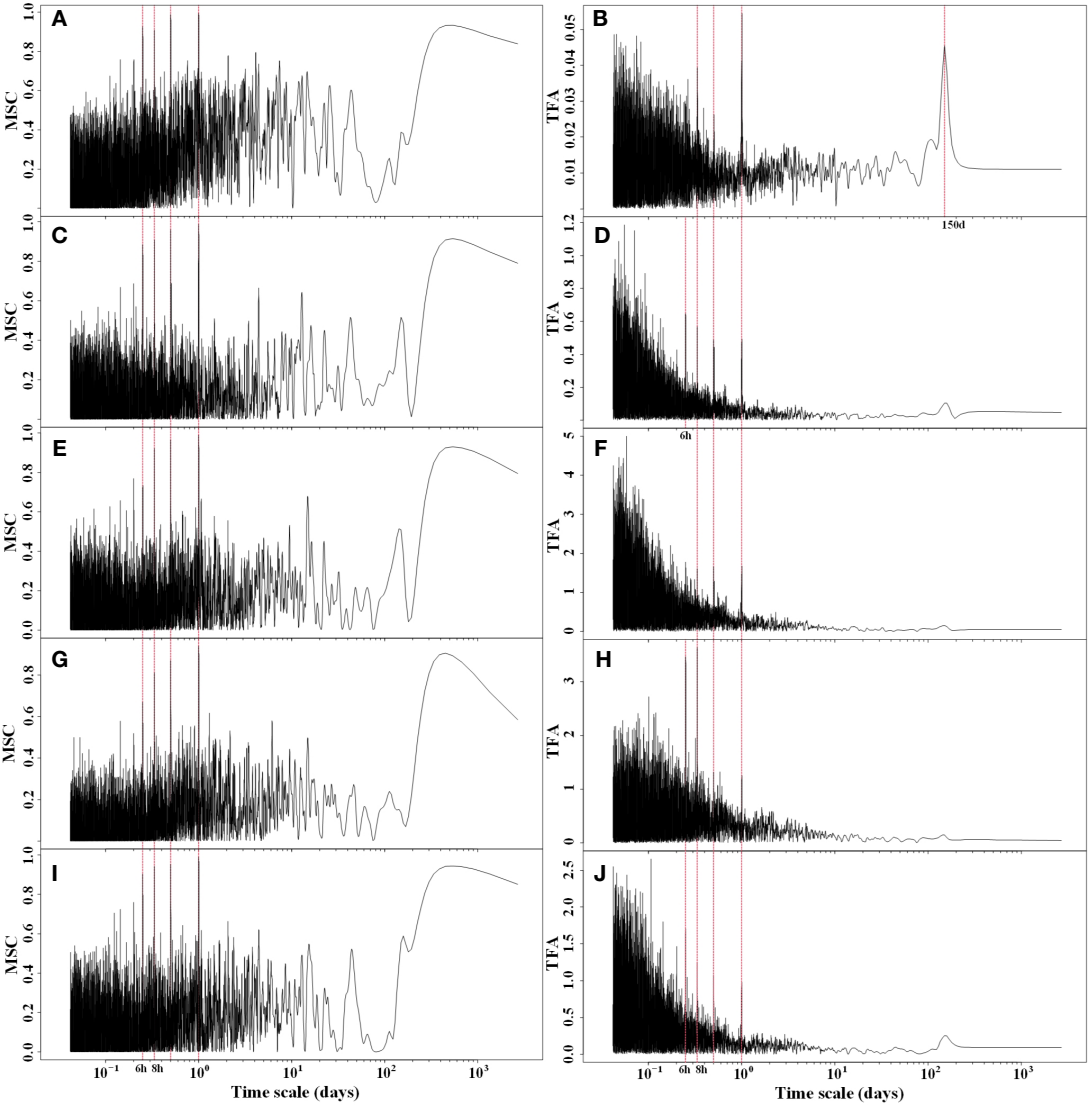
Magnitude squared coherence (MSC) and transfer function amplitude (TFA) between half-hourly NEP and **(A, B)** Rg, **(C, D)** RH, **(E, F)** Ta **(G, H)** Ts, and **(I, J)** VPD.

After conducting cross-spectral analysis of Rg, RH, Ta, Ts, and VPD with NEP, it was evident that these five meteorological factors exhibited a strong coherence with NEP (**Figure 4**). This coherence was particularly pronounced for long periods of 12 hours, 1 day, and 1 year, while it was relatively weaker for short periods. The cross-spectral analysis of meteorological factors with NEP partially explained the multiple periodic variation patterns of NEP. The transfer function amplitudes of these meteorological factors and NEP all displayed variation periods of 6 hours, 8 hours, 12 hours, and 1 day. Additionally, the greatest magnitude of the MSC was observed at the 1 d period. This implies that there is a strong synchronous variation of NEP with meteorological factors, especially at the daily scale. Therefore, the circadian rhythm of NEP in the Tugai forest was regulated by meteorological conditions.

**Figure 5:**
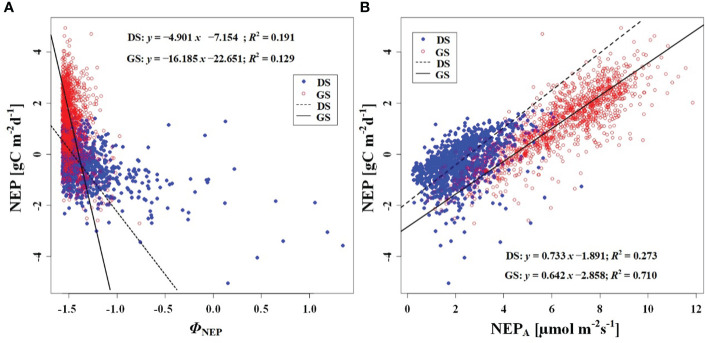
Relationship between **(A)** NEP and phase of NEP (*Φ*_NEP_), and **(B)** between NEP and NEP_A_. DS and GS indicate the dormant and growing seasons, respectively.

**Figure 6:**
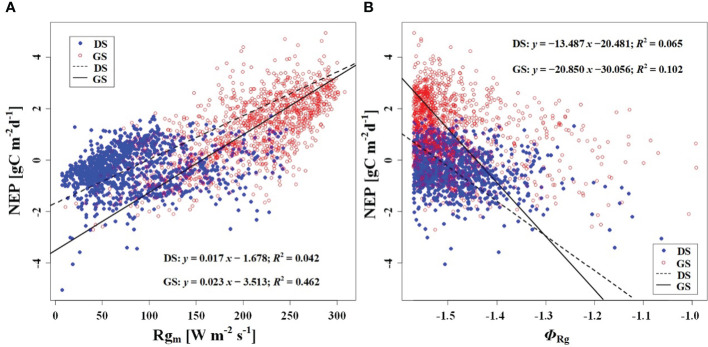
Relationship **(A)** between NEP and Rg_m_, and **(B)** between NEP and phase of Rg (*Φ*_Rg_). DS and GS indicate the dormant and growing seasons, respectively.

In order to investigate the response of CO_2_ uptake capacity in the desert Tugai forest ecosystem to changes in the meteorological conditions, we conducted an analysis of the relationship between daily NEP and meteorological factors. Our findings suggested the *Φ*_NEP_ and *Φ*_Rg_ demonstrated negative correlations with daily NEP both during the dormant and growing season (**Figures 5A**, **6B**). Conversely, the NEP_A_ and Rg_m_ significantly positively correlated with daily NEP (**Figures 5B**, **6A**). This indicated that the higher the asymmetry in the diurnal changes of NEP, the higher the daily NEP value, as a result of a higher peak in diurnal NEP.

**Figure 7:**
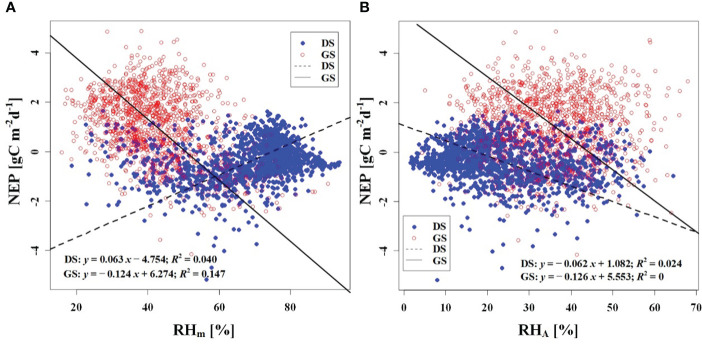
Relationship **(A)** between NEP and RH_m_, and **(B)** between NEP and RH_A_. DS and GS indicate the dormant and growing seasons, respectively.

**Figure 8:**
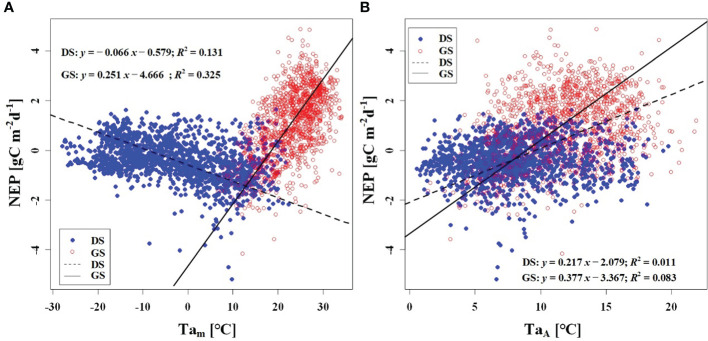
Relationship **(A)** between NEP) and Ta_m_, and **(B)** between NEP and Ta_A_. DS and GS indicate the dormant and growing seasons, respectively.

**Figure 9:**
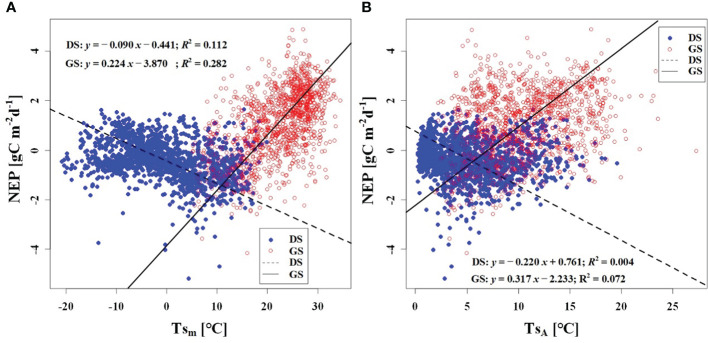
Relationship **(A)** between NEP and Ts_m_, and **(B)** between NEP and Ts_A_. DS and GS indicate the dormant and growing seasons, respectively.

**Figure 10:**
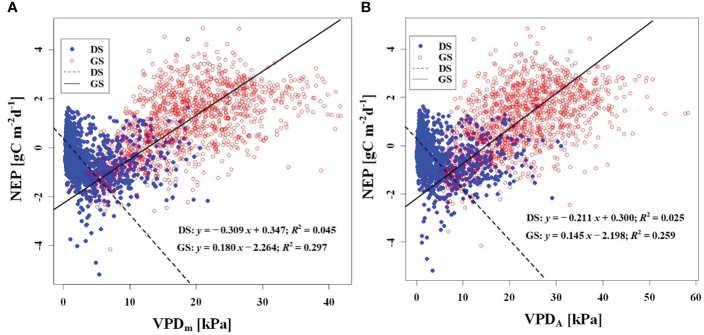
Relationship **(A)** between NEP and VPD_m_, and **(B)** between NEP and VPD_A_. DS and GS indicate the dormant and growing seasons, respectively.

We observed a weak negative correlation between the RH_m_ and daily NEP in the growing season (*R* ^2^ = 0.147), but no significant correlation was found in the dormant season (**Figure 7A**). In contrast to RH_m_, Ta_m_, Ts_m_, and VPD_m_ exhibited weak positive correlations with NEP in the growing season, while weak negative correlations were observed in the dormant season (**Figures 8A**–**10A**). The RH_A_ showed no significant correlation with NEP in both the growing and dormant seasons (**Figure 7B**). The significant effect of the VPD_A_ changes on NEP during the growing season suggested that asymmetric variations in diurnal VPD significantly affect the carbon uptake of Tugai forest ecosystems (**Figure 10B**).

In this study, the analysis of seasonal differences in the correlation between NEP and driving factors unveiled significant seasonal variations in NEP’s response to meteorological factors (likelihood ratio > 26 and *P*< 0.001, see **Supplementary Material**). The seasonal difference in magnitude and direction of response were observed. This implied that there were seasonal differences in the synchronization between NEP and meteorological factors at the daily scale.

### 3.3 The impact of meteorological variations on net ecosystem production

**Figure 11:**
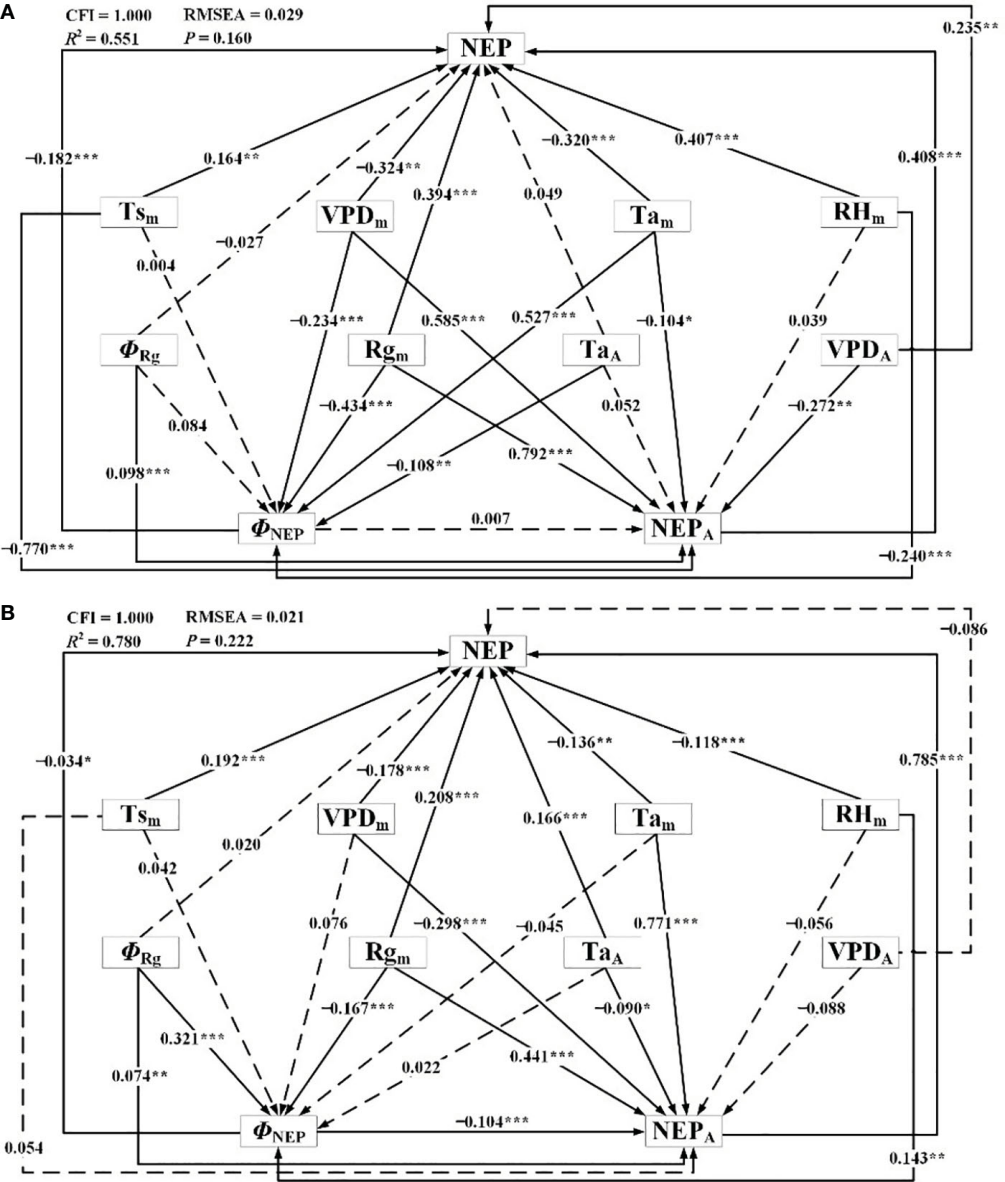
Structural equation model representing connections between net ecosystem productivity and meteorological factors during **(A)** the dormant and **(B)** growing season. *Φ*_NEP_: phase of diurnal NEP, NEP_A_: amplitude of diurnal NEP, Ts_m_: daily mean Ts, Ta_m_: daily mean Ta, Ta_A_: amplitude of diurnal Ta, Rg_m_: daily mean Rg, *Φ*_Rg_: phase of diurnal Rg, RH_m_: daily mean RH, VPD_m_: daily mean VPD, VPD_A_: amplitude of diurnal VPD. ^*^ *P*<0.10, ^**^ *P*<0.05, and ^***^ *P*<0.01.

The results of the SEM demonstrating the influence of meteorological factors on NEP during the dormant and growing seasons were presented in **Figure 11**; **Table 4**. Through correlating NEP with the influencing factors, the models were meticulously fitted and tested to establish statistically significant direct and indirect effects while gradually eliminating statistically insignificant pathways. For the dormant season, the fitted *R* ^2^ was 0.551, the RMSEA was 0.029 (*P* = 0.160), and the CFI was 1.000. In the growing season, the corresponding values were 0.780, 0.021 (*P* = 0.222), and 1.000, respectively. Overall, the results indicated that both models were suitable, with the structural equation model for the growing season slightly outperforming that for the dormant season.

**Table 4:** Direct and indirect effects of meteorological factors on NEP during the dormant and growing season.

Factors	Dormant season			Growing season		
	Direct effect	Indirect effect	Total effect	Direct effect	Indirect effect	Total effect
*Φ*_NEP_	−0.182^***^	0.003	−0.179^***^	−0.034	−0.081^***^	−0.116^***^
NEP_A_	0.408^***^		0.408^***^	0.785^***^		0.785^***^
Ts_m_	0.164^**^	−0.315^***^	−0.151^***^	0.192^***^	0.047	0.239^***^
Ta_m_	−0.320^***^	−0.142^***^	−0.457^***^	−0.136^**^	0.610^***^	0.474^***^
Ta_A_	0.049	0.040^*^	0.090^**^	0.166^***^	−0.073^*^	0.093^*^
Rg_m_	0.394^***^	0.401^***^	0.795^***^	0.208^***^	0.365^***^	0.573^***^
*Φ*_Rg_	−0.027	0.025^*^	−0.002	0.020	0.021	0.041
RH_m_	0.407^***^	0.059^**^	0.466^***^	−0.118^***^	−0.061^*^	−0.179^***^
VPD_m_	−0.324^**^	0.281^***^	−0.043	−0.178^***^	−0.243^***^	−0.421^***^
VPD_A_	0.235^**^	−0.111^**^	0.124	−0.086	−0.069	−0.155^**^

Φ_NEP_, phase of diurnal NEP; NEP_A_, amplitude of diurnal NEP; Ts_m_, daily mean Ts; Ta_m_, daily mean Ta; Ta_A_, amplitude of diurnal Ta; Rg_m_, daily mean Rg; Φ_Rg_, phase of diurnal Rg; RH_m_, daily mean RH; VPD_m_, daily mean VPD; VPD_A_, amplitude of diurnal VPD. ^*^P<0.10; ^**^P<0.05; and ^***^P<0.01.

In the SEM, during the dormant season, Ta_m_, Rg_m_, RH_m_, VPD_m_, and Ta_A_ had significant direct effects on *Φ*_NEP_. In contrast, during the growing season, *Φ*_Rg_, Rg_m_, and RH_m_ had significant direct effects on *Φ*_NEP_ (*P*< 0.10, in descending order of relative importance). For NEP_A_ during the dormant season, Rg_m_, Ts_m_, VPD_m_, VPD_A_, and Ta_m_ had significant direct effects, while during the growing season, Ta_m_, Rg_m_, VPD_m_, *Φ*_NEP_, Ta_A_, and *Φ*_Rg_ had significant direct effects (*P*< 0.10, in descending order of relative importance). The Ta_A_ significantly influenced the *Φ*_NEP_ in Tugai forests during the growing season, with no notable impact on the NEP_A_. However, this pattern may be inverted during the dormant season, the Ta_A_ could affect NEP_A_ without influencing Φ_NEP_. Notably, both during the dormant and growing seasons, Ts_A_ had no statistically significant direct effect on *Φ*_NEP_ (*P* > 0.10), RH_m_ had no statistically significant direct effect on NEP_A_, and *Φ*_Rg_ had no statistically significant direct effect on daily NEP. Rg_m_ had significant indirect effects on daily NEP through the NEP_A_ and *Φ*_NEP_ during both the dormant and growing seasons (*P*< 0.10). This underscored the critical roles that the amplitude and phase of diurnal NEP played in regulating the light response of NEP.

In addition to the direct effects of *Φ*_NEP_ on daily NEP during both the dormant and growing seasons, the *Φ*_NEP_ impacted daily NEP by reducing the NEP_A_ during the growing seasons, resulting in significant negative total effects on NEP (*P*< 0.05). This suggested that regulating the phase of ecosystem circadian rhythms reduced the daily NEP by lowering its peak of net carbon uptake. It’s worth noting that the total effects of *Φ*_Rg_ on daily NEP were not statistically significant (*P* > 0.1). Seasonal differences were observed in the effect directions of RH_m_ on daily NEP, with direct effects being predominant during both the dormant and growing seasons. The effects of VPD_m_ on daily NEP were primarily indirect during the growing seasons. Reductions in the VPD_A_ may lead to increased daily NEP in Tugai forests during the growing season. Conversely, this relationship may be reversed during the dormant season. The VPD_A_ exerted a negative indirect effect on daily NEP through NEP_A_ during both the dormant and growing seasons. This indirect effect counterbalanced the positive direct effect of the changing amplitude in water vapor on daily NEP during the dormant season but intensified during the growing season. Daily NEP was more sensitive to the changes in VPD_A_ than to the variations in Ta_A_. This finding suggests that the daily-scale variation in VPD has a more pronounced impact on the daily NEP within the ecosystem than the daily-scale fluctuations in air temperature.

The results of the SEM analysis comparing the dormant and growing seasons revealed that an increase in NEP_A_ led to an increase in daily NEP during both seasons. Conversely, an increase in the *Φ*_NEP_ contributed to a decrease in NEP_A_, resulting in decreased daily NEP. Reductions in the Ta_A_ may lead to decreased daily NEP in Tugai forests during both the growing and dormant season. During the dormant season, an increase in Ta_A_ indirectly led to an increase in daily NEP by negatively affecting *Φ*_NEP_, while during the growing season, the increase in VPD_A_ caused a decrease in NEP. This suggested that regulated the daily NEP during the dormant season, while water availability limited the daily NEP during the growing season. The variation in the indirect and direct effects of daily changes in meteorological factors highlighted the seasonal differences in the mechanisms by which the NEP of Tugai forests influenced meteorological conditions, as well as differences in the response to various meteorological factors. The indirect effects of daily changes in meteorological factors through NEP_A_ and *Φ*_NEP_ on daily NEP indicated that the diurnal variability in meteorological conditions influenced the daily NEP of Tugai forests by regulating the circadian rhythm of this ecosystem.

## 4 Discussion

### 4.1 Response of NEP to variations in meteorological conditions at different time scales

Ecosystem carbon exchange has been reported to exhibit daily, weekly, and monthly cycles of variability (Baldocchi et al., 2001; Stoy et al., 2005). Consistent with these established findings, our own observations similarly revealed periodic variations in NEP (**Figure 3**). The diurnal NEP fluctuations are likely attributable to the photoperiod resultant from the Earth’s rotation. The discerned 11-day and 14-day NEP periodic variations might potentially find explanation in the seiches of Ebinur Lake, as analogous courses of variation were evident in the meteorological factors, particularly in RH (**Figure 3**). The periodic variations in NEP and meteorological factors highlight the complex interactions between lake dynamics and meteorological conditions in shaping the carbon exchange dynamics of the desert riparian forest ecosystems. Remarkably, this phenomenon has been identified for the first time in our study. Consequently, we advocate for an integrated approach in investigations of ecosystem carbon exchange within regions influenced by lakes, necessitating the incorporation of the lakes’ impact on local climate.

Within the Tugai forest, we have observed a striking and significant synchronization between NEP and meteorological conditions, underscoring the prominent influence of these conditions on ecosystem carbon exchange. The MSC and TFA between NEP and meteorological factors in the Tugai forest suggested that NEP has the robust synchronization with meteorological conditions on the daily scale. The findings suggested a close alignment with the light response of ecosystem carbon exchange at daily scale (**Figure 4**). It’s worth noting that prior studies have illuminated the presence of a desert-oasis effect in the desert riparian forests (Li et al., 2016; Teng et al., 2021), which significantly influences the daily variability of meteorological conditions. This effect further bolsters the synchronization observed between NEP and meteorological factors on a daily scale. Similarly, there was high synchronization between NEP and temperature at daily scale. In addition, there was a significant response of NEP in Tugai forest to mean daily temperature and its daily variation during both growing and dormant seasons. The reason is that temperature plays a crucial role in ecosystem carbon exchange as it significantly influences metabolism (Anderson-Teixeira et al., 2011). In arid and hot regions, high temperatures intensify evaporation and reduce water availability for plants, impairing carbon sequestration of trees by reducing photosynthesis and increasing respiration (Adams et al., 2009). Additionally, sustained elevation in high temperatures accelerates respiration rate and causes enzyme denaturation, further hindering the carbon sequestration capacity of plants (Banbury Morgan et al., 2021).

The response of NEP to meteorological conditions is intricate due to the differential sensitivities of plant leaves, roots, and soil microorganisms. Leaves exhibit increased photosynthesis with rising temperature, while respiration is regulated by stomatal conductance, resulting in a dynamic and adaptive response. During the dormant season, we observed weak correlations between NEP and daily average meteorological factors. However, during the growing season, NEP in the Tugai forest exhibited high sensitivity to daily variations in RH (**Figure 7**). This sensitivity arises from short-term changes in canopy RH that often coincide with thickening cloud cover or rainfall, leading to reduce solar radiation availability and, subsequently, decreased net CO_2_ uptake. These findings suggest that there were significant differences in response mechanisms of the Tugai forest ecosystem carbon exchange to changing meteorological conditions at various time scales.

In this study, we observed significantly diurnal variations in meteorological conditions and NEP of the desert riparian forest. The diurnal variations in light, temperature, and humidity regulate the circadian rhythms of plant physiological processes and rhizosphere microbial communities, leading to synchronous changes in photosynthesis and respiration, which in turn strongly correlate with NEP at the daily scale (Gil and Park, 2019). Thus, we conclude that meteorological factors remained the primary drivers of ecosystem carbon exchange of the desert riparian forest at short time scales, both half-hourly and daily, which is consistent with previous studies (López-Blanco et al., 2017; Ma et al., 2017). It should be noted that the synchronization of meteorological factors with NEP does not follow a linear pattern across various time scales (**Figure 3**). Overall, the findings contribute to understanding the meteorological influences on NEP in the desert riparian forest ecosystem, highlighting the significance of light, temperature, and humidity dynamics at different time scales.

### 4.2 The effects of meteorological conditions on NEP at daily scale

Ecosystem carbon exchange is composed of two fundamental components: photosynthesis and ecosystem respiration. It’s important to note that forest canopy photosynthesis does not adhere to a linear relationship with absorbed effective solar radiation. Moreover, its sensitivity to solar radiation is recognized to be influenced by the proportion of diffuse radiation. As a result, exercising caution becomes imperative when attempting to employ uncomplicated linear light-response models to replicate photosynthesis across both temporal and spatial scales (Running et al., 1999; Heinsch et al., 2006). Daily integration of the independent and dependent variables on a daily basis can effectively linearize the complex relationship between half-hourly photosynthesis and light (Leuning et al., 1995). This process of linearization significantly bolsters the SEM model of NEP response to meteorological factors that has been constructed within the framework of this study.

As a temperate desert riparian forest, the ecosystem respiration and photosynthesis exhibited significant variations in response to temperature fluctuations. Moreover, the NEP in the Tugai forest was dominated by gross primary productivity (GPP) during the growing season and by ecosystem respiration (R_eco_) during the dormant season, leading to notable seasonal variations in NEP with temperature. When the daily Ta rises and the Ta_A_ decreases, the increase in nighttime Ta was greater than the increase in daytime Ta (Thorne et al., 2016). This asymmetric warming leads to increasing soil microbial activity, soil organic carbon decomposition, and ecosystem carbon emissions, resulting in a decrease in NEP (Xia et al., 2014). This is consistent with the response of NEP to temperature in this study. The inverse relationships between temperature (including atmospheric and soil temperature) and NEP in different seasons (**Figures 8**, **9**) further demonstrated the seasonal variations in carbon exchange processes in the Tugai forest ecosystem. The direct effect of atmospheric temperature on NEP was seasonally distinct (dormant season was larger than growing season), but both exhibited a significant negative effect; whereas the indirect effect was not only seasonally disparate (dormant season was larger than growing season), but also displayed an opposite pattern (**Table 4**). The main reason for the differences in NEP response to temperature under different seasons is the different ecosystem processes that dominate CO_2_ exchange. During the dormant season, CO_2_ exchange in the Tugai forest ecosystem was primarily driven by carbon emissions from ecosystem respiration. During the growing season, when the temperature increases, soil respiration in the Tugai forest ecosystem was augmented along with ecosystem photosynthesis. Due to the Kok effect, the respiration rate of plant leaves decreased during the daytime, resulting in an increase in NEP (Kok, 1948, Kok, 1949; Yin et al., 2020).

Ecosystem-scale indirect meteorological forcings play a crucial role in shaping the NEP over the long term. One of the key factors contributing to this indirect effect is the sensitivity of the NEP to diurnal fluctuations in temperature and humidity, especially when plants are exposed to diverse temperature and humidity regimes. In this study, we identified temperature as the main limiting factor for NEP in the Tugai forest during the dormant season. Increasing temperatures enhanced ecosystem respiration, resulting in a decrease in ecosystem carbon uptake. Conversely, during the growing season, VPD emerged as the primary limiting factor for NEP. Although VPD had no direct significant effect on NEP in this study, it exerted an indirect significant influence (**Table 4**). The Tugai forest, located near riverbanks within an arid desert zone, experiences a warm growing season, often exceeding the optimal temperature for photosynthesis. Simultaneously, VPD levels typically surpass the optimal threshold, thereby regulating leaf stomata and inhibiting carbon uptake. On one hand, under favorable environmental conditions characterized by stability and less variability in meteorological factors, plants allocate more resources to growth and carbon assimilation, leading to an increase in net carbon uptake. On the other hand, unfavorable environmental conditions with unstable and highly variable meteorological factors can pose challenges to plants. In such conditions, plants may experience temperature stress and limited water availability, resulting in a higher rate of forward and reverse biochemical reactions (McDowell et al., 2008). These conditions can even lead to the denaturation of enzymes, ultimately reducing carbon uptake.

The intricate interplay between alterations in plant physiology and fluctuations in diurnal Ta and VPD contributes to the observed indirect effects on NEP. A comprehensive understanding of these complex interactions involving Ta and VPD is pivotal for comprehending the responses of ecosystem carbon exchange to environmental drivers. This study emphasizes the importance of accounting for the indirect effects of rising diurnal Ta and VPD when analyzing the seasonal variation of NEP in the desert riparian forest.

### 4.3 The uncertainty analysis and future prospects

In this study, we measured the NEP using the open path eddy covariance system. It is well known that the sensor-path heat exchange (SPHE) and analyzer temperature sensitivity reduce the ability of the open path eddy covariance system to characterize the response of ecosystem carbon exchange to radiative forcing (Burba et al., 2008). The effect of SPHE cannot be completely eliminated using current correction methods, such as the WPL correction, especially during the growing season (Deventer et al., 2021). Therefore, despite the use of the WPL correction in this study, the effect of SPHE may have a potential impact on the response of forest NEP to the changing solar radiation, during both the growing and dormant seasons. Notably, due to stable atmospheric conditions or non-stationary high-frequency time series, gaps in EC- flux measurements are unavoidable and require gap filling. Current gap filling methods are all based on the changing characteristics of the time series of flux measurements and its response mechanism to meteorological factors. This study employed a fully data-based machine learning method to gap filling, preserving as much as possible the changing pattern of the flux data and its response to meteorological factors. These aspects are beyond the scope of this study and will be investigated in future studies.

Forest NEP is subject to synergistic or antagonistic effects of several factors, such as microclimate, stand age, understory species composition, phenology, and disturbance patterns. The principal factor determining the seasonal variation of all CO_2_ exchange fluxes (NEP, GPP, and R_eco_) is the phenology of the understory vegetation, and plant community structure also plays a major role in the variation of CO_2_ exchange fluxes. Vegetation phenology regulates leaf development, and the larger the leaf area, the higher the light absorption capacity. Consequently, the CO_2_ uptake by photosynthesis is also increased. Ecosystem respiration has a strong correlation with GPP, and studies have confirmed the strong influence of vegetation productivity on R_eco_ (Janssens et al., 2001). As ecosystems alter structurally and functionally over time, possibly in response to disturbances, predicting changes in ecosystem function will become increasingly important (Richardson et al., 2007). Forests are more productive and susceptible to natural and anthropogenic disturbances (e.g., deforestation, fire, grazing, etc.), whereas the Ebinur Lake watershed is relatively pristine and has existed for thousands of years in its present undisturbed oasis form. Tugai forests in the Ebinur Lake basin may have evolved self-regulatory mechanisms that have contributed to their endurance through various meteorological regimes over time (including seasonal and interannual variability in the area of Ebinur Lake waters) (Yang et al., 2014; Wang et al., 2019). It also benefits from the protective policy implemented within the national nature reserve, which ensures that the groundwater depth of our site in the Ebinur Lake basin is consistently maintained at less than 3 m (Yang et al., 2014). Notably, if the groundwater depth surpasses 6 m, the Tugai forests face a heightened risk of decline due to the impacts of climate change (Zhou et al., 2020). If functional changes in ecosystem responses result from adjustments to long-term exposure to specific average meteorological conditions, then in the short term such as 1–5 years, the direct effects of meteorological changes can dominate changes in CO_2_ fluxes (Teklemariam et al., 2010). As the protection of the Ebinur Lake watershed is enhanced and the lake area expands, the functional type of plants across the Ebinur Lake watershed may alter in the future, with an increased prevalence of shrubs and trees, which will change the CO_2_ fluxes in the area.

The potential for climate- or weather-induced changes in ecosystem response functions underscores the need to interpret measured NEP models through more than just parallel comparisons with driving meteorological conditions. This is because such an analysis would only capture transient dependencies between variables (Teklemariam et al., 2010). This point is supported by the correlation analysis between NEP and meteorological factors for different seasons, which indicates that NEP measured during the growing season exhibits the strongest correlation with the meteorological conditions at the time of flux measurements (**Figure 11**). Therefore, it is essential to also consider the environmental and ecological history of the site, including the adaptations that have taken place over time.

## 5 Conclusions

This study aimed to investigate the variations in NEP of a representative desert riparian forest ecosystem across multiple temporal scales, elucidating its relationship with meteorological conditions. Utilizing approximately seven years of eddy covariance flux measurements, we conducted a comprehensive examination of the cycles exhibited by the NEP and meteorological conditions. These cycles spanned various lengths, ranging from five hours to one year. The NEP variations exhibited a robust correlation and synchronization with meteorological conditions across diverse temporal scales, with the most significant fluctuations occurring in one-day cycle. Over the seven-year duration of our research, these findings also revealed significant seasonal differences in the direct and indirect responses of the NEP to the averages, amplitudes, and phases of diurnally changing meteorological factors at a daily scale. These variations included the differences in the magnitude of response and even a reversal of the response directions. The amplitude of diurnal air temperature significantly influenced the phase of diurnal NEP in Tugai forests during the growing season, with no notable impact on the amplitude of diurnal NEP. However, this pattern may be inverted during the dormant season. These findings underscore the substantial impact of circadian rhythms induced by meteorological conditions on the NEP of desert riparian forests at an ecosystem scale. Given the significance of both the direct and indirect effects, as well as the amplitude of periodic meteorological factors variations, careful consideration is essential when assessing the ecosystem carbon exchange of desert riparian forests. The extended time frame allows for a more comprehensive analysis of dynamics and strengthens the robustness of our conclusions. These findings provide valuable insights into the complex responses of desert riparian forests to climate change, thereby contributing to the advancement of our scientific understanding of carbon exchange pattern within arid and semi-arid ecosystems.

## Data availability statement

The raw data supporting the conclusions of this article will be made available by the authors, without undue reservation.

## Author contributions

DT: Formal analysis, Funding acquisition, Visualization, Writing – original draft. XG: Formal analysis, Methodology, Validation, Writing – review & editing. XH: Data curation, Investigation, Methodology, Writing – review & editing. JW: Funding acquisition, Software, Validation, Writing – review & editing. GL: Conceptualization, Investigation, Supervision, Writing – review & editing. JW: Data curation, Formal analysis, Software, Validation, Writing – review & editing. XY: Formal analysis, Investigation, Resources, Supervision, Validation, Writing – review & editing.

## Glossary

EC: eddy covariance
GDD: growing degree day
IMF: intrinsic mode function
NEE: net ecosystem CO_2_ exchange
NEP: net ecosystem productivity
NEP_A_: amplitude of diurnal net ecosystem productivity
MSC: magnitude-squared coherence
Rg: global solar radiation
Rg_m_: daily average of global solar radiation
RH: relative humidity
RH_m_: daily average of relative humidity
RH_A_: amplitude of diurnal relative humidity
SEM: structural equation model
Ta: air temperature
Ta_m_: daily average of air temperature
Ta_A_: amplitude of diurnal air temperature
TFA: transfer function amplitude
Ts: soil temperature
Ts_m_: daily average of soil temperature
Ts_A_: amplitude of diurnal soil temperature
VPD: vapor pressure deficit
VPD_m_: daily average of vapor pressure deficit
VPD_A_: amplitude of diurnal vapor pressure deficit
*Φ*_NEP_: phase of diurnal net ecosystem productivity
*Φ*_Rg_: phase of diurnal Rg
